# Profiling the Antidiabetic Potential of Compounds Identified from Fractionated Extracts of *Entada africana* toward Glucokinase Stimulation: Computational Insight

**DOI:** 10.3390/molecules28155752

**Published:** 2023-07-30

**Authors:** Sunday Amos Onikanni, Bashir Lawal, Valens Munyembaraga, Oluwafemi Shittu Bakare, Muhammad Taher, Junaidi Khotib, Deny Susanti, Babatunji Emmanuel Oyinloye, Lloyd Noriega, Ayodeji Famuti, Adewale Oluwaseun Fadaka, Basiru Olaitan Ajiboye

**Affiliations:** 1 College of Medicine, Graduate Institute of Biomedical Sciences, China Medical University, Taichung 40402, Taiwan; u109305125@cmu.edu.tw; 2 Department of Chemical Sciences, Biochemistry Unit, Afe-Babalola University, Ado-Ekiti 360101, Ekiti State, Nigeria; babatunjioe@abuad.edu.ng; 3 Department of Pathology, University of Pittsburgh, Pittsburgh, PA 15213, USA; bal140@pitt.edu; 4 Institute of Translational Medicine and New Drug Development, College of Medicine, China Medical University, Taichung 40402, Taiwan; u111314104@cmu.edu.tw; 5 University Teaching Hospital of Butare, Huye 15232, Rwanda; 6 Department of Biochemistry, Faculty Science, Adekunle Ajasin University, Akungba Akoko 342111, Ondo State, Nigeria; oluwafemi.bakare@aaua.edu.ng; 7 Department of Pharmaceutical Technology, Kulliyyah of Pharmacy, International Islamic University Malaysia, Kuantan 25200, Pahang, Malaysia; mtaher@iium.edu.my; 8 Pharmaceutics and Translational Research Group, Kulliyyah of Pharmacy, International Islamic University Malaysia, Kuantan 25200, Pahang, Malaysia; 9 Department of Pharmacy Practice, Faculty of Pharmacy, Airlangga University, Surabaya 60115, Indonesia; 10 Department of Chemistry, Kulliyyah of Science, International Islamic University Malaysia, Kuantan 25200, Pahang, Malaysia; deny@iium.edu.my; 11 Biotechnology and Structural Biology (BSB) Group, Department of Biochemistry and Microbiology, University of Zululand, KwaDlangezwa 3886, South Africa; 12 Institute of Drug Research and Development, SE Bogoro Center, Afe Babalola University, PMB 5454, Ado-Ekiti 360001, Ekiti State, Nigeria; basiru.ajiboye@fuoye.edu.ng; 13 Honey T Scientific Company, Ibadan 234002, Oyo State, Nigeria; famutiayodeji@gmail.com; 14 Department of Biotechnology, University of the Western Cape, Bellville 7535, South Africa; afadaka@uwc.ac.za; 15 Phytomedicine and Molecular Toxicology Research Laboratory, Department of Biochemistry, Federal University, Oye-Ekiti 371104, Ekiti State, Nigeria

**Keywords:** glucokinase, *Entada africana*, pharmacophore, simulation, hydrophobic

## Abstract

Glucokinase plays an important role in regulating the blood glucose level and serves as an essential therapeutic target in type 2 diabetes management. *Entada africana* is a medicinal plant and highly rich source of bioactive ligands with the potency to develop new target drugs for glucokinase such as diabetes and obesity. Therefore, the study explored a computational approach to predict identified compounds from *Entada africana* following its intermolecular interactions with the allosteric binding site of the enzymes. We retrieved the three-dimensional (3D) crystal structure of glucokinase (PDB ID: 4L3Q) from the online protein data bank and prepared it using the Maestro 13.5, Schrödinger Suite 2022-3. The compounds identified were subjected to ADME, docking analysis, pharmacophore modeling, and molecular simulation. The results show the binding potential of the identified ligands to the amino acid residues, thereby suggesting an interaction of the amino acids with the ligand at the binding site of the glucokinase activator through conventional chemical bonds such as hydrogen bonds and hydrophobic interactions. The compatibility of the molecules was highly observed when compared with the standard ligand, thereby leading to structural and functional changes. Therefore, the bioactive components from *Entada africana* could be a good driver of glucokinase, thereby paving the way for the discovery of therapeutic drugs for the treatment of diabetes and its related complications.

## 1. Introduction

The challenges of diabetes mellitus have been linked to persistent hyperglycemia (high level of blood glucose), polydipsia, and polyphagia, thereby resulting in insulin deficiency or malfunctioning [1,2,3]. The global surge of this disease requires a multi-targeted approach with the statistics revealing a 10.5% incidence rate worldwide in 2022, which could rise above 12.5% by 2030 if drastic steps are not taken to address this challenge [3]. In 2020, approximately four million deaths were recorded globally on account of diabetes patients due to the rapid increase in those incidence cases associated with both lifestyle changes and environmental factors [4,5].

Indisputably, important successes have been recorded in the management of diabetes with conventional hypoglycemic agents based on acclaimed antidiabetic properties studied in different laboratories across the world, especially in developing countries [6,7]. However, limitations exist in several ways such as a decrease over time in potency, high cost, and the proliferation of side effects [8]. These concerns have led scientists in search of better and safer routes, leading to involvement of the medicinal drug approach and development that could combat this menace [9,10].

Furthermore, the use of herbal medicine is now widespread throughout the world and is well-recognized today because over 1500 plants possess antidiabetic potency, and these medicinal plants have been confirmed to have normoglycemic properties [11,12]. One good example of these medicinal plants is *E. africana*. This plant belongs to the family of Fabaceae and grows mostly in parts of Sub-Saharan African countries with a savannah location. The plant is locally used to cleanse wounds, stoppage of bleeding, diuretic application, anti-inflammatory, and anti-gonococci [11,13,14]. The plant has been identified to contain varieties of bioactive compounds as well as nutritional components such as proteins, starch, dietary fiber, and fat [15,16]. Moreover, the bioactive compounds reported in recent research studied by Adewole et al. (2022) included betulinic acid, betulin, robinetin, myricetin, apigenin, naringin, and naringenin. Myricetin is a known nutraceutical value plant-derived flavonoid with strong potency in anti-oxidant, antidiabetic and anti-inflammatory activities [17], apigenin possesses anti-inflammatory potential with the ability to improve testosterone, protect against cancer, and boost the quality of sleep [18,19], while robinetin shows an antiperoxidative potential when tested in rat liver microsomes induced by CCl_4_ where surprisingly, robinetin was shown to be a potent antiperoxidative agent with an IC_50_ value [20].

Moreover, computational prediction in drug targeting in recent studies have become an important tool in drug design and the development of drug molecules for the management of human diseases. This approach has clear procedures that have significantly shortened drug discovery and contributed to computational pharmacology and therapeutics [21,22]. Several mechanistic approaches have been unveiled through computational simulation to understand the molecular mechanism of chemical agents before testing in an animal model [22]. Among these computational methods are pharmacophores, homology models, auto-quantitative structure–activity relationship (auto-QSAR) prediction, and absorption, distribution, metabolism, excretion, toxicity (ADMET). Computational modeling prediction of the ligand affinity for molecular targets in diabetes has been widely studied [23,24]. The inhibitory potential of compounds from the alliaceous plant protein tyrosine phosphatase 1B (PTP1B) has been predicted in silico as a possible mechanism of antidiabetic effects [25,26]. Glucokinase (GK) is an essential enzyme in glucose metabolism and a potential therapeutic target in type 2 diabetes [27]. The glucokinase enzyme is a principal regulator of glucose homeostasis within the liver and pancreatic cells [28,29]. Therefore, this enzyme suffices as an ideal drug target, especially in type 2 diabetes, because its activation reduces the blood sugar level [29].

Despite many recent advances in managing diabetes mellitus and its associated complications through the research on phytochemicals of *E. africana*, less focus has been placed on the molecular level with glucokinase as a way of understanding a few of its mechanisms of action and identifying its antidiabetic agents. Therefore, the identified *E. africana* compounds were subjected to molecular docking analysis against glucokinase (PDB ID: 4L3Q) to identify compounds from the plant for antidiabetic drug discovery.

## 2. Results

**Figure 1:**
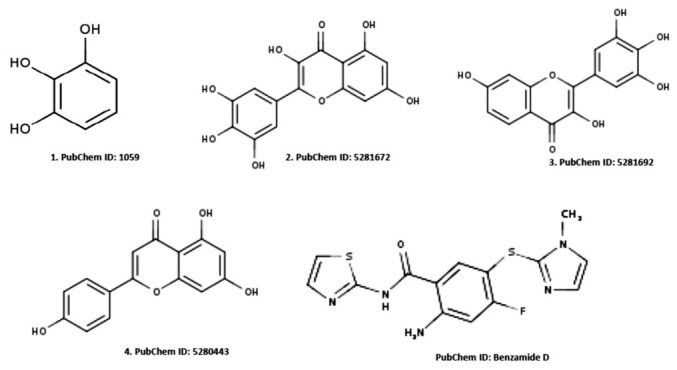
Structural details of the identified active ligands (**1**,**2**, **3**, and **4**) and benzamide D as the reference.

**Figure 2:**
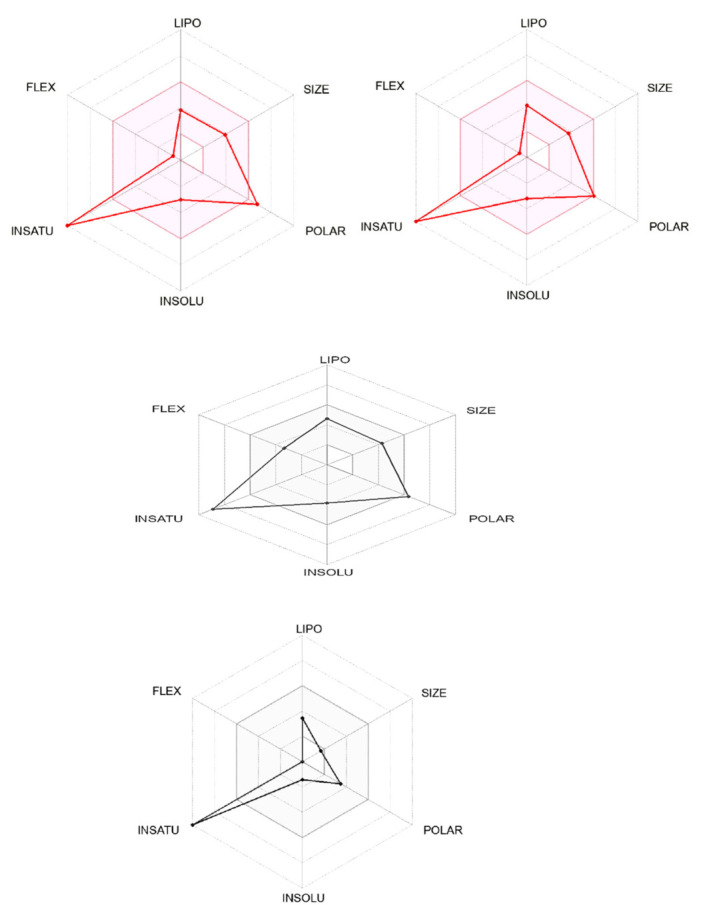

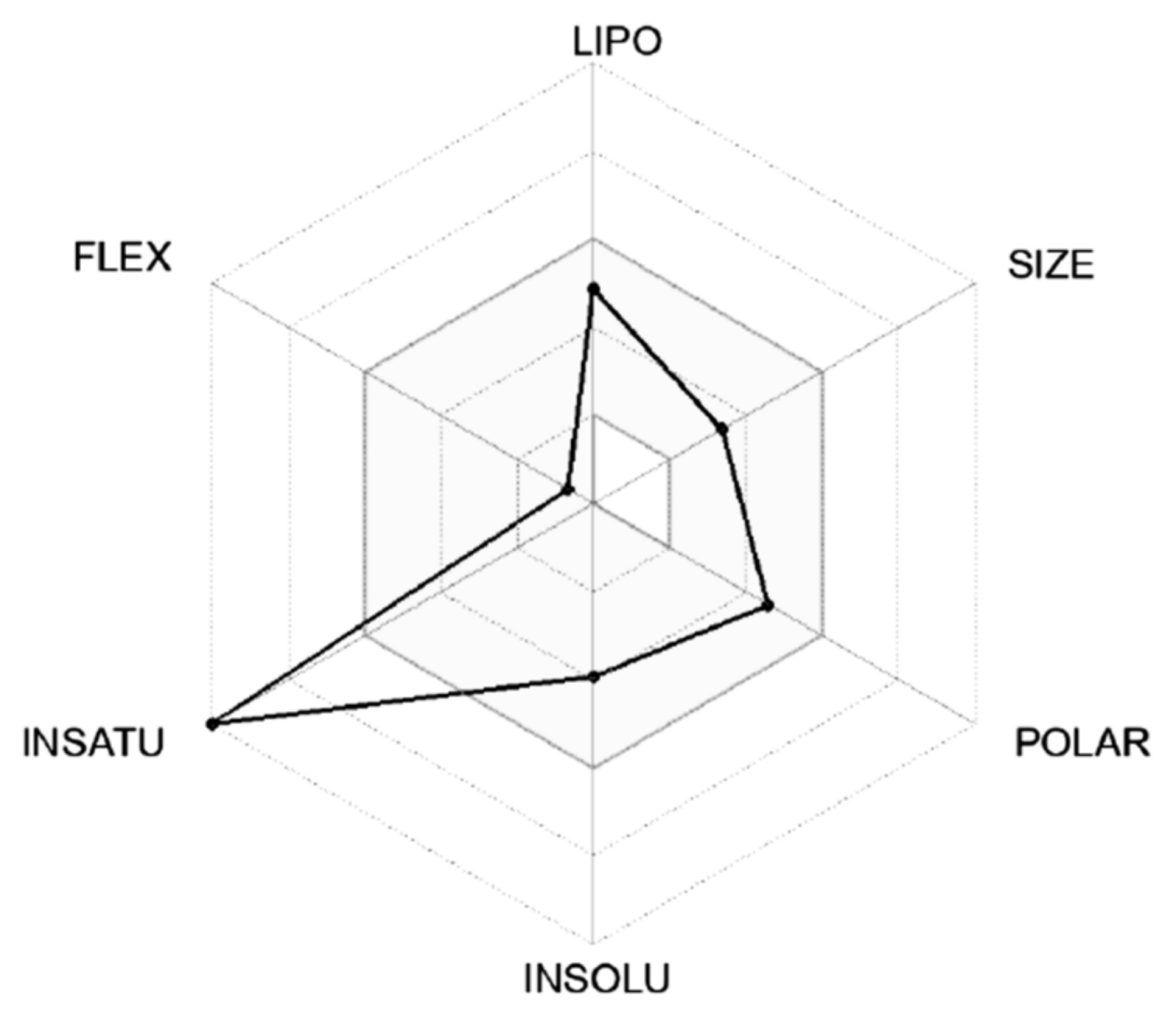
The colored zone of the identified ligands from *Entada africana* shows the suitable physicochemical space for oral bioavailability.

The acceptability of the hydrogen bonds of the solute when reacted with the different molecules at any given points could be related to the number of hydrogen bonds that would be donated by the solute to the water molecules in an aqueous phase. Therefore, the structural composition of the identified ligands when compared to the standard ligand is shown in Figure 1. Furthermore, the Figure 2 revealed the suitable physicochemical space for oral bioavailability of identified compounds which could be compared favorably with the standard compound. Surprisingly, our ligands showed a successfully processed molecule on the predicted central nervous system activity ranging between −1 and −3 when compared with the standard (as shown in Table 1).

**Table 1:** The target protein with the physicochemical properties of the docked ligands from the fractionated extract of *Entada africana*.

PubChem ID	MW	CNS	donorHB	accptHB	dip^2/V	# Acid
**1057**	126.032	−1	3	2.25	0.0184394	0
**5281672**	318.038	−2	5	6	0.0087447	0
**5281692**	302.043	−2	5	6.25	0.0242827	0
**5280443**	270.053	−2	2	3.75	0.0556372	0
**Benzamide D**	349.047	−1	2	2.25	0.0050193	0

Note: accptHB: acceptor hydrogen bond; CNS: central nervous system; dip^2/V: square of the dipole moment divided by molecular volume; donorHB: donor hydrogen bond; MW: molecular weight; # Acid: number of carboxylic acids; (**1057**: pyrogallol; **5281672**: myricetin; **5281692**: robinetin; **5280443**: apigenin).

Furthermore, the ability of any compound to comply with the famous Lipinski rule of five always resulted in showing poor pharmacokinetic potential. Therefore, our results indicate that all of the examined parameters were within the Lipinski’s rule of five (ROF) cutoff range for the test ligands, with the potential of having no violation of Lipinski’s ROF and the Veber rules, as shown in Table 2.

Our study also compared the inhibitory potential ligands from the fractionated extract of *Entada africana* using SWISSADME, as shown in Table 3. The study indicated that the ligands of interest (5281672 and 5281692) exerted no inhibition following the Pgp substrate compared with the standard (benzamide D), an inhibitor of the Pgp substrate. Surprisingly, one of the lead compounds from the fractionated extract of *Entada africana* produced low gastrointestinal absorption when compared with the standard ligand, thereby indicating that most ligands showed an ATP-dependent drug efflux pump for ADME compounds with substantial potency through substrate specificity.

Drug-likeness outputs and the medicinal importance from the research with receptor binding and cleavage domain in the State 1 receptor complex with few ligands revealed amazing results, as shown in Table 4.

Furthermore, with relevant molecules already predicting the target of interest by docking analysis, a similar event was unraveled with the use of the fractionated extract of *Entada africana* with the crystal structure domain of the glucokinase-activator complex 5281672 and 5281692, producing a competitive docking score of −8.297 kcal/mol and −6.42 kcal/mol, respectively, when compared with the standard docking score of −5.259 kcal/mol (Table 5).

**Table 4:** Drug-likeness output and medicinal chemistry of the test ligands from *Entada africana*.

PubChem	Lipinski # Violations	Ghose # Violations	Veber # Violations	Egan # Violations	Muegge # Violations	Bioavailability Score	Pains # Alerts
**5281672**	1	0	1	1	2	0.55	1
**5281692**	0	0	0	0	0	0.55	1
**5280443**	0	0	0	0	0	0.55	0
**Benzamide D**	0	0	0	1	0	0.55	0
**1057**	0	3	0	0	1	0.55	1

**Table 5:** Interaction of the docking results of the complexes.

TG	Compd	Dock Score	MMGBSA	# H-Bond	Pi-Cat	Salt Bridges
**4L3Q**	**5281672**	−8.297	−44.39	2(GLU256), GLU290, ASN204, THR168,THR228, GLY229	0	0
	**5281692**	−6.42	−34.84	ASP78, GLY81, THR228, GLY229, GLU290, GLU256	0	0
	**5280443**	−1.784	−33.09	ASP409	0	0
	**Benzamide D**	−5.259	−51.55	LYS229, ASP205	0	ASP409, ASP78
	**1057**	−7.215	−34.02	THR168, GLU256, 2(ASN204), ASP205	LYS169	0

Note: 4L3Q, crystal structure of glucokinase-activator complex; (**1057**: pyrogallol; **5281672**: myricetin; **5281692**: robinetin; **5280443**: apigenin).

**Figure 3:**
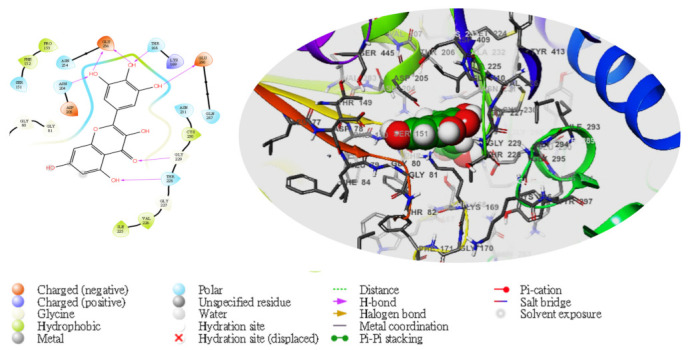
The molecular complexes of the glucokinase-activator interacted at the active site with **5281672** (the green stick model). The 2D (**left**) and 3D (**right**) with the negative, positive, and neutral charges of the binding site residues are represented in the red, blue, and white colors, respectively.

**Figure 4:**
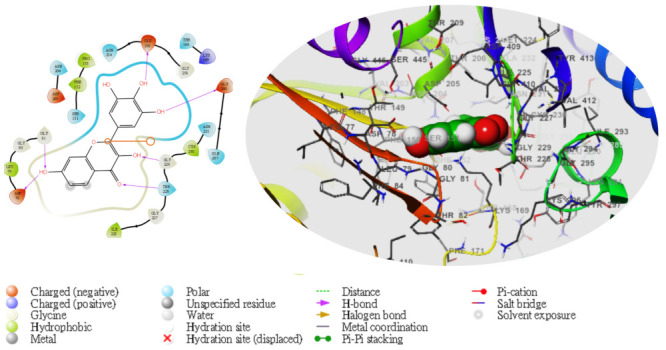
The molecular complexes of the glucokinase-activator interacted at the active site with **5281692** (the green stick model). The 2D (**left**) and 3D (**right**) with the negative, positive, and neutral charges of the binding site residues are represented in the red, blue, and white colors, respectively.

**Figure 5:**
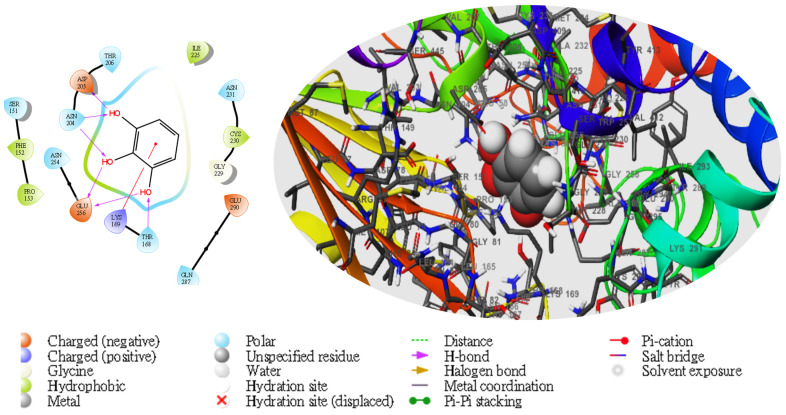
The molecular complexes of the glucokinase-activator interacted at the active site with **1057** (the green stick model). The 2D (**left**) and 3D (**right**) with the negative, positive, and neutral charges of the binding site residues represented by the red, blue, and white colors, respectively.

**Figure 6:**
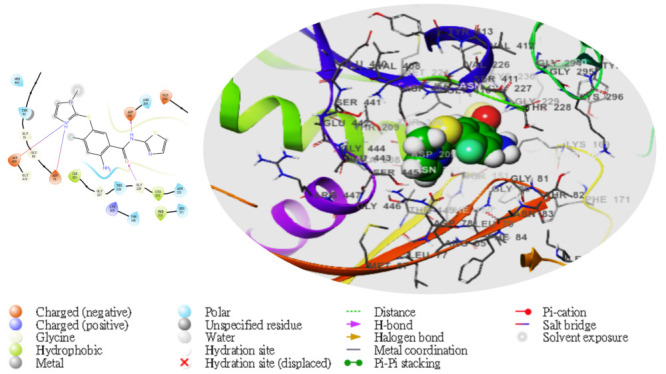
The molecular complexes of the glucokinase-activator interacted at the active site with **benzamide D** (the green stick model). The 2D (**left**) and 3D (**right**) with the negative, positive, and neutral charges of the binding site residues are represented with red, blue, and white colors, respectively.

**Figure 7:**
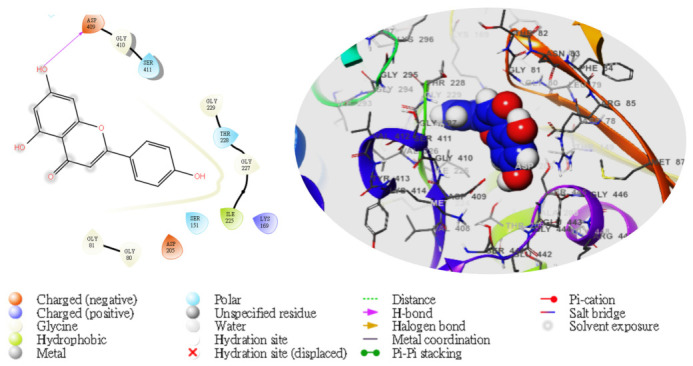
The molecular complexes of the glucokinase-activator interacted at the active site with **5280443** (the green stick model). The 2D (**left**) and 3D (**right**) with the negative, positive, and neutral charges of the binding site residues represented with the red, blue, and white colors, respectively.

Moreover, following the specificity of the interactions, the ASP205 in the N-terminal domain receptor was involved in hydrogen bonding with the atoms showing no π–π interaction, but a salt bridge was formed by ASP409 and ASP78 with the glucokinase-activator complex, whereas GLU256, GLU290, ASN204, and THR168 were involved in hydrogen bonding in the N-terminal domain receptor with 5281672, while THR228, GLY229, ASP78, GLY81, THR228, GLY229, GLU290 formed the same as the N-terminal domain receptor with 5281692. However, GLU256, ASN204, ASP205 were involved in hydrogen bonding in the N-terminal domain receptor with the 1057 ligand with LYS169 at the π–cation interaction, as indicated in Table 5. The overall interaction revealed good measurable binding affinities for the receptor residues with ligand contribution to the flexibility of the target. Therefore, in the molecular docking and analysis, both 2D and 3D, respectively, among the top two ligands that formed, the target receptor showed that the top two ligands of interest could occupy the active site of the enzyme (Figure 3, Figure 4, Figure 5, Figure 6 and Figure 7).

## 3. Discussion

One of the ground-breaking options in the last decades has been the use of bioactive molecules from medicinal plants for the amelioration and management of challenges caused by diseases including diabetes mellitus and its related complications [8,30,31]. Surprisingly, many of these medicinal plants have been known for its medicinal value in the treatment of human diseases such as diabetes mellitus and obesity [11,32,33]. Importantly, one of these medicinal plants is *E. africana*, which is known for its vulnerability and ability to cleanse wounds and stop bleeding [15,34,35]. Most parts of western Africa consume the leaves and stem bark as a tonic and anti-inflammatory source [15,36]. However, less information is available on the anti-diabetic potential of the fractionated action of some phytocompounds present in *E. africana*, which could be a pathway to the development of novel drugs for the treatment of diabetes mellitus and its complications. Therefore, a target-based computational approach was explored on compounds identified in the fractionated extract from our previous research for its anti-diabetic potential [16]. Our research focused on glucokinase (which has a vital role in glucose metabolism and as a rate limiting enzyme in the development of type 2 diabetes) as a molecular target for the identification of anti-diabetic components among the identified compounds [28]. Therefore, the results obtained from our research revealed four promising phytocompounds from *E. africana* (1057: pyrogallol; 5281672: myricetin; 5281692: robinetin; 5280443: apigenin) that possess some structural features and molecular interactions required for them to be considered for further experiments toward GK activation and glucose regulation. The prediction of ligands to have an H-bond donor (HBD) less than or equal to five and an H-bond acceptor (HBA) less than or equal to ten with accurately processed molecules on the estimated central nervous system activity of +2 has been suggested in various research studies. Violation of a single molecular property of the above control could lead to absorption and bioavailability (Lipinski, 2002). Interestingly, the parameters of the studied molecules fit the ROF cutoff region and did not violate Lipinski’s rule, demonstrating the most ideal pharmacological potency. Additionally, the SWISSADME results, as shown in Table 3, demonstrated that the ligands 5281672 and 5281692, which were expected to be Pgp substrate inhibitors, exhibited no inhibition after the Pgp substrate analysis as opposed to the standard (benzamide D).

When compared to benzamide D, bioactive molecules from *Entada africana*’s fractionated extract resulted in gastrointestinal absorption. This revealed that most ligands exhibit a substrate-specific ATP-dependent drug efflux pump for ADME drugs with significant efficacy. Furthermore, the ability of any potential therapeutic molecule to transverse the bi-layer and escape the blood–brain barrier may be attributed to oral bioavailability. Among these functions are desolvation, diffusion, resolvation, and physicochemical properties including lipophilicity (Malathi et al., 2019). The dimensions provided by drug distribution and excretion can be used to identify pharmacokinetic models. It is therefore essential to investigate the pharmacokinetic properties of any bioactive molecule or potential therapeutic drugs prior to consideration during drug development (MacGregor, J.T., et al.,2001). Therefore, the main two test ligands showed a permeability effect on the human intestinal membrane and blood–brain barrier. However, to validate the pharmacological potential of the main ligands, the relevant molecules predicted the target of interest by docking analysis, and a similar event was unraveled with the use of the fractionated extract of *Entada africana* with the crystal structure domain of the glucokinase-activator complexes 5281672 and 5281692, producing a competitive docking score of −8.297 kcal/mol and −6.42 kcal/mol, respectively, when compared with the standard docking score of −5.259 kcal/mol (Table 5).

Furthermore, the results from the computational analysis showed that three ligands—pyrogallol, myricetin, and robinetin—revealed a better binding affinity when compared with the referenced compound benzamide D, a potent glucokinase activator (GKA) with a competitive docking score, as shown in Table 5. Therefore, the modulatory effect of the protein–ligand complex revealed via the docking score (Table 5) indicates that the fractionated *E. africana* could possibly modulate glucokinase activity [37,38]. Furthermore, Molecular Mechanics-Generalized Born expanse (MMGBSA) has been scientifically adopted worldwide to validate the results of molecular docking analysis for its binding capacity, and the stability of the receptor–ligand interactions thereby prove the binding affinity score on the predicted level to the specificity and binding strength of the ligand in the binding pocket of the said receptor [39,40,41,42]. Therefore, the free energy of binding (∆G Bind) of the two important compounds ranged from −34.84 to −44.39 kcal/mol, as shown in Table 5, indicating strong attraction between the ligand–receptor interaction from the research.

Furthermore, Molecular Dynamics studies (MDs) were performed to show the atomic level movement and changes in the said receptor for 400 based on timescale. To determine the potency of the identified ligands as an important activator of the receptor, the use of Schrodinger_2022-1 was explored to examine the stability of the protein’s active site to perform the MDs on the receptor and in complex with the ligands. Most of the properties of the MDs including molecular surface area (MolSA), root mean square deviation (RMSD), root mean square fluctuation (RMSF), and solvent-accessibility surface area (SASA) were analyzed.

**Figure 8:**
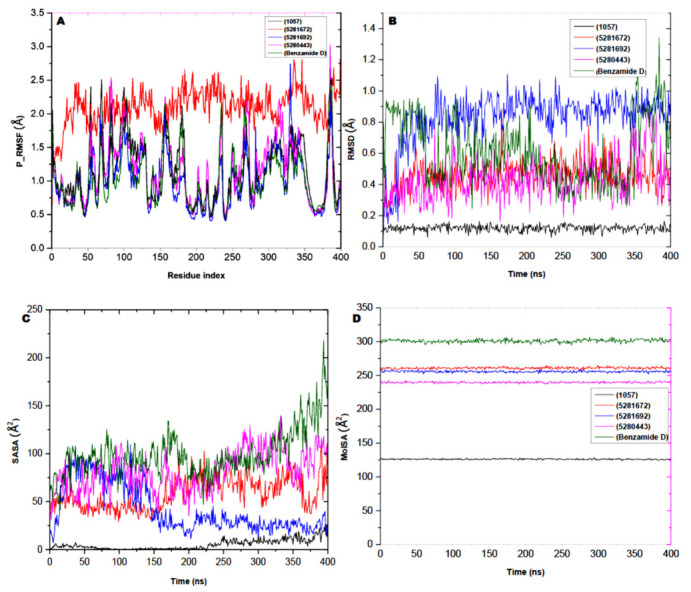
Analysis of the Molecular Dynamics (MD) of the glucosidase activator-complexed with (**1057**: pyrogallol; **5281672**: myricetin; **5281692**: robinetin; **5280443**: apigenin). (**A**) P_-_RMSF graphical illustration, (**B**) a graph of RMSD, (**C**) SASA plot, and (**D**) MolSA diagram. All data related to Molecular Dynamics (MD) were performed via Schrödinger version 2022_1 software version.

Moreover, to gain insights into the effect of ligand–receptor interaction for the flexibility and stability, the root mean square deviation (RMSD) and root mean square fluctuation (RMSF) were plotted for their interaction with the molecules, as shown in Figure 8A,B, respectively. The rGyr and SASA were plotted to analyze the molecular surface area (MolSA) and solvent-accessibility surface area (SASA) to find the compactness and compute the secondary structure of the complexes, respectively (Figure 8C,D). However, the interpretation of the structural stability of the protein alongside the complex and molecules were interpreted using deviation of the plot and the mean ± SEM, as shown in Table 6. Based on the results obtained from our study from the simulation time of 400 ns, there were alterations in the RMSD patterns at 25–70 ns, after which all of the ligand showed equilibrium until the end of the simulation period. By analyzing the mean ± SEM of the plots, the receptor-robinetin had the highest RMSD value (0.82 Å) while the receptor-standard (0.61 Å) appeared to be next when compared to the receptor-myricetin (0.45 Å). The receptor-pyrogallol (0.11 Å) had the lowest RSMD value. Data from this study indicate variations in the mean deviation, which explains the behavior of the protein–ligand complexes under biological conditions, and thus provides a platform for further research. To further study the dynamism of the protein residues and their behavior, the root mean square fluctuation (RMSF) pattern was studied with the mean fluctuation between ligands and the standard compound as shown in Table 6. Similar to the RMSD results obtained, the fluctuation patterns of the complexed molecules when compared with RMSF showed relative value with the standard compound. The highest mean fluctuations were observed when complexed with myricetin (2.09 Å), followed by receptor-apigenin (1.14 Å). The receptor-robinetin (1.00 Å) competed with while the receptor-standard (1.05 Å) had a similar value.

Furthermore, with the importance of surface area surrounding the solvent molecules, the solvent-accessibility surface area (SASA) was studied to find out the protein interaction and surface molecules to provide an insight into the protein folding ability for its stability and structural formation, conformation, and rearrangement. The results of the deviation value plotted are shown in Table 6 and in Figure 8C. Therefore, the standard complex had a higher mean SASA value of 99.18 Å, while both the receptor-robinetin and receptor-myricetin had mean SASA values of 58.40 Å and 42.40 Å, respectively. However, receptor-apigenin showed a high mean SASA value of 81.10 Å compared with the other ligands. The distribution of amino acid residues from either the accessible area or the buried region may be linked to the observed SASA variation. This could contribute or impact the structural conformation of the complexes. To further investigate the effects of the rearrangement of amino acid residues from either the accessible region or the buried region, the complexes of the receptor and ligands were subjected to molecular surface area (MolSA) analysis. For the ligands, there was a constant value within the range of 126–300 Å with reduced fluctuations throughout the simulation period, as shown in Table 6. The deviation value from the plotted data showed that the standard-complex had a higher mean value of 301.05 Å, which was stable from the onset and throughout the 400 ns simulation period while one of the lead compounds (receptor-robinetin) competed as well with a mean value of 260.94 Å. Pattern fluctuation was observed at the beginning of the simulation time until the end of the simulation period. However, the receptor-myricetin showed a pattern of fluctuation of 255.76 Å with no noticeable drop in pattern from the original pattern position until the end of the simulation time. Conclusively, ligands 5281672 and 5281692 indicated minor pattern variation when complexed with the receptor in contrast to benzamide D. All of these results show that the studied molecules complexed with glucosidase demonstrated high stability.

## 4. Materials and Methods

### 4.1. Protein Preparation

This study obtained the proteins of interest specifically retrieved from the Research Collaboratory for Bioinformatics Database (http://www.rcsb.org/pdb) assessed on 21 March 2023, followed by the removal of the co-crystallized ligands. Next, the protein preparation wizard Maestro 13.5, Schrödinger Suite 2022-3 by Glide was used to assign bond ordering, and hydrogen bonds were added to form disulfide bonds, while Prime was used to fill in missing side chains and loops. Next, the structure was reduced using OPLS2005 and optimized using PROPKA after water molecules beyond 3.0 of the heteroatoms were eliminated [21,39,43]. Finally, the receptor grid file was created to define the ligand-binding pocket.

### 4.2. Ligand Preparation

A compound library of twenty (20) from the fractionated extracts of *Entada africana* with a small molecular weight identified based on the previous study was further examined in silico-wise [44]. The ligand preparation was carried out based on LigPrep, the latest module of Maestro 13.5, Schrödinger Suite 2022-3, followed by OPLS3 at a physiological pH of 7.2 ± 0.2. The possible ionization states for each ligand structure were generated. Each ligand’s stereoisomers were calculated by keeping certain chiralities constant while varying other [39].

### 4.3. Receptor Grid Generation

For the ligand docking analysis, receptor grid generation allows for the determination of the position and size of the protein’s active region. Using the receptor grid construction tool in Maestro 13.5, Schrödinger Suite 2022-3, the scoring grid was defined supported by the ligand glucokinase (PDB ID: 4L3Q). The van der Waals (vdW) radius scaling factor for the nonpolar receptor atoms was set to 1.0, with a partial charge cutoff of 0.25 to further lower the potential of the receptor’s nonpolar. 

### 4.4. Molecular Docking Analysis

The receptor grid file result was further utilized to perform molecular docking investigations with the Glide Maestro 13.5, Schrödinger Suite 2022-3. Standard precision (SP) was employed to dock the protein glucokinase, and therefore the prepared ligands from *E. africana*, along with the quality activator keeping the ligand sampling, were set to flexible and ligand sampling was set to none (refine only). For the ligand atoms, the vdW radius scaling factor was scaled at 0.80 with a partial charge cutoff of 0.15.

### 4.5. Predictions of ADME Properties and Toxicological Potential 

The features of the test drugs’ absorption, distribution, metabolism, excretion, and toxicity (ADMET) were assessed using in silico integrative model predictions of the lead compounds with the SWISSADME and Qikprop Maestro 13.5, Schrödinger Suite 2022-3.

### 4.6. Binding Free Energy Calculation

The Prime Molecular Mechanics-Generalized Born area MM-GBSA tool of Maestro 13.5, Schrödinger Suite 2022-3 is accustomed to determining the steadiness of the protein–ligand complexes according to their binding free energy. The ligands were prepared beforehand using LigPrep, and the relevant proteins were prepared using the protein preparation wizard, as detailed by the MM-GBSA technology available with Prime [45]. Sitemap anticipated the active sites of the proteins. Glide standard precision (SP) docking was then accustomed to dock the chemicals with proteins. The MM-GBSA technology offered with Prime was utilized to find the binding free energy of the ligand–protein complexes utilizing the Prime MM-GBSA panel. The OPLS3 physical phenomenon was chosen, and therefore the continuum solvent model was VSGB. The default settings for the opposite options were selected.

### 4.7. Analysis of MD Simulations and Trajectory Point

Molecular Dynamics (MD) simulation of the target receptor (4L3Q) was prepared via the Maestro 13.5, Schrödinger Suite 2022-3 with Maestro version 13.1.137, MM share version 5.7.137, and Windows-x64 Platform. Following the previous research by [46,47], analysis of the buildup system was followed by the MD preparation and trajectory module. The adopted option of the Desmond module with the force field OPLS 2005 was used for the MD docked to each. Each of the identified protein–ligand complexes was bounded with a predefined transferable intermolecular potential with a 3-point water model in an orthorhombic box. The addition of sodium and chloride ions helps the physiological condition to be determined after abrogation of the whole charge was reduced. The use of the Nose–Hoover thermostat helped constantly maintain the temperature at 310 °C and a Martyna–Tobias–Klein barostat (United States) maintained pressure at 1.01325. The simulation relaxation was undertaken with the use of the NPT ensemble after considering the number of atoms, the pressure, and the timescale. During the MD simulation, the particle mesh Ewald method was used to determine the long-range electrostatic, MD simulation was carried out for 400 ns, and the trajectory sampling was set at an interval of 100 ps with 1000 frame numbers. The simulation outputs were analyzed and visualized by a simulation interaction diagram and an MS-MD trajectory analysis. The replicate of the MD simulated was carried out to avoid variation and all the data were plotted by using OriginPro version 9.

## 5. Conclusions

The growing concern toward diabetes and its complications calls for serious attention as it will have a devastating effect on mankind if not approached seriously. The herein identified hit candidates possessed binding affinities and peculiar molecular interactions with glucokinase, thereby showing some satisfactory pharmacokinetic properties and did not violate Lipinski’s ROF. Therefore, they could be further developed as glucokinase activators for the possible treatment of diabetes and its related complications. The compounds should be subjected to lead-optimization and experimental studies for possible development as anti-diabetic drugs.

## Figures and Tables

**Table 2:** Bio-absorbability output compounds from the fractionated extract of *Entada africana*.

PubChem ID	HOA	% HOA	SAfluorine	ROF	ROT	PSA
**1057**	2	73.94	0	0	0	65.396
**5281672**	2	28.18	0	1	1	162.033
**5281692**	2	49.831	0	0	1	138.922
**5280443**	3	73.877	27.655	0	0	100.61
**Benzamide D**	3	93.431	0	0	0	87.867

Note: HOA, human oral absorption; PSA, polar surface area; ROF, rule of five; ROT, rule of three, SAfluorine: solvent-accessible surface area of fluorine atoms.

**Table 3:** Pharmacokinetics and medico-toxicological analyses of the identified molecules. The docking was performed with SWISSADME, while the medico-toxicological effect was performed with the Pred-hERG and AdmetSAR online servers.

PubChem	GI Abs	BBB-p	Pgp-S	CYP1A2-I	CYP2C19-I	CYP2C9-I	CYP2D6-I	CYP3A4-I
**5281672**	Low	No	No	Yes	No	No	No	Yes
**5281692**	High	No	No	Yes	No	No	Yes	Yes
**5280443**	High	No	No	Yes	No	No	Yes	Yes
**Benzamide D**	Low	No	No	Yes	Yes	Yes	Yes	Yes
**1057**	High	Yes	No	No	No	No	No	Yes

Note: BBB-p, blood–brain barrier permeant; GI abs, gastrointestinal absorption; I, inhibitor; S, substrate; -, negative.

**Table 6:** MD properties of the native protein and protein–ligand interactions.

Compound	RMSD	RMSF	MolSA	SASA
**5281672**	0.45 ± 0.09	2.09 ± 0.28	260.94 ± 1.33	58.40 ± 16.27
**5281692**	0.82 ± 0.17	1.00 ± 0.40	255.76 ± 1.24	42.40 ± 24.85
**5280443**	0.43 ± 0.13	1.14 ± 0.46	239.68 ± 1.08	81.10 ± 20.46
**Benzamide D**	0.61 ± 0.20	1.05 ± 0.42	301.05 ± 2.23	99.18 ± 24.42
**1057**	0.11 ± 0.02	1.12 ± 0.44	126.20 ± 0.75	513.30 ± 5.12

Note: Values are represented as the mean ± SEM measured in Armstrong unit (Å).

## Data Availability

Not applicable.

## Sample Availability

Samples of the compounds are/are not available from the authors.
